# Rural LGBTQIA+ Aging in New England: Demand for Service Connection and Social Supports

**DOI:** 10.1093/geroni/igaf122.1732

**Published:** 2025-12-31

**Authors:** Breana Bietsch

**Affiliations:** Bridgewater State University, Glastonbury, Connecticut, United States

## Abstract

In the New England region of the United States, approximately 5% of people aged 18 and over identify as Lesbian, Gay, Bisexual, Transgender, Queer, Intersex, and Asexual (LGBTQIA+) (Conron & Goldberg, 2020), with no statistics specific to LGBTQIA+ people aged 50 and over or who live in a rural area of this region. This demographic information is much needed to better understand the extent of the health needs for rural LGBTQIA+ adults, as rural compared to urban LGBTQIA+ adults often face worse health outcomes, lack access to health care, have limited community supports, experience social isolation, and discrimination (McKay et al., 2024). In the current study, which asked participants in focus groups (n = 33) and individual interviews (n = 15) about their lived experiences regarding what impacts their health, participants echoed these concerns of rural, older LGBTQIA+ adults. Themes of Lack of Services and Attachments and Social Connections showed that rural areas of New England were fundamentally worse off compared to participants in urban settings. For example, rural participants spoke about limited access to affirming LGBTQIA+ services, lack of health care service coordination, and sparse social connection opportunities. Participants suggested solutions for services to be more interconnected, receive attention for innovation, and LGBTQIA+ aging competent. Participants also called for more positive, face-to-face programming that allowed for mentorship within LGBTQIA+ communities, such as intergenerational programs or congregate meals. Implications of the current study point to building programs in rural areas that are more interconnected, affirming, and increase mentorship opportunities to improve health.

